# Randomised controlled decentralised feasibility trial of a fixed low-dose combination antihypertensive drug strategy to attenuate cognitive decline in high-risk adults

**DOI:** 10.1136/bmjopen-2023-080862

**Published:** 2024-08-24

**Authors:** Cheryl Carcel, Lauren Clancy, Katie Harris, Ruth Peters, Aisling Byrne, Kimberley Bassett, Ruth Freed, Camilla M Hoyos, Anthony Rodgers, Richard Lindley, John Chalmers, Ying Xu, Mark Woodward, Menglu Ouyang, Sharon L Naismith, Craig Anderson

**Affiliations:** 1 The George Institute for Global Health, Faculty of Medicine, University of New South Wales, Sydney, New South Wales, Australia; 2 Department of Neurology, Royal Prince Alfred Hospital, Camperdown, New South Wales, Australia; 3 Neuroscience Research Australia, Randwick, Sydney, New South Wales, Australia; 4 Brain and Mind Centre, The University of Sydney, Camperdown, New South Wales, Australia; 5 Westmead Applied Research Centre, University of Sydney, Sydney, New South Wales, Australia; 6 The George Institute for Global Health, Imperial College London, London, UK; 7 The George Institute for Global Health China, Beijing, China

**Keywords:** Blood Pressure, Dementia, Clinical Trial

## Abstract

**Objectives:**

The Action To promote brain HEalth iN Adults study aimed to determine the feasibility and applicability of recruitment using home blood pressure (BP) monitoring, routine blood biochemistry and videoconference measures of cognition, in adults at high risk of dementia.

**Design:**

A decentralised double-blind, placebo-controlled, randomised feasibility trial with a four-stage screening process.

**Setting:**

Conducted with participants online in the state of New South Wales, Australia.

**Participants:**

Participants were aged 50–70 years with moderately elevated BP (systolic >120 and <160 mm Hg or diastolic >80 and <95 mm Hg) and ≥1 additional enrichment risk factor of monotherapy treatment of hypertension, diabetes mellitus, elevated low-density lipoprotein cholesterol, obesity, current smoking or a first degree relative with dementia, which indicated an elevated risk for future cognitive decline.

**Intervention:**

Triple Pill (active antihypertensive treatment of telmisartan 20 mg, amlodipine 2.5 mg and indapamide 1.25 mg) or placebo Triple Pill (blinded study capsules).

**Primary and secondary outcome measures:**

Primary outcome was feasibility of the study expressed as the percentage of participants randomised from those who were screened. Secondary outcomes were the applicability of videoconference measures of cognition and the overall trial, tolerability of the Triple Pill, safety outcomes and medication adherence.

**Results:**

The proportion (95% CI) of patients randomised to those screened was 5% (2%–10%). The applicability of the trial expressed as percentage of those who completed all remote assessments over the number of randomised participants was 67% (95% CI 05 to 22%). There were no serious adverse events or withdrawals from treatment. All participants adhered to study medication, except for one person who had two capsules left at the end of the study period.

**Conclusions:**

The feasibility of this decentralised trial on BP lowering in patients at high risk for dementia is low. However, the applicability of remote assessments of cognitive function is acceptable.

**Trial registration number:**

Australian New Zealand Clinical Trials Registry (ANZCTR): ACTRN12621000121864.

**STRENGTHS AND LIMITATIONS OF THIS STUDY**

- This feasibility study was conducted in a rigorous manner with detailed screening and reporting processes.
- The results are limited by the sample size and homogeneity (participants were English-speaking white Caucasian adults).
- The scale-up ability and generalisability of this study may be limited.

## Introduction

 Because we have an ageing population, the absolute numbers of those experiencing dementia and cognitive decline are increasing. A new case of dementia is estimated to be diagnosed every three seconds in the world, and total numbers are predicted to rise to 152 million by 2050, 68% of people with dementia will be residing in low and middle-income countries.^1 2^ Dementia has a long prodrome with the pathology arising decades before the onset of symptoms, and modifiable cardiovascular (CV) risk factors such as hypertension, diabetes and smoking, all acting to increase the future risk.^3^,^5^ As treatments to alter the neurodegenerative course of dementia are expensive, targeted towards only one of the pathological drivers of dementia (Alzheimer’s disease) and of modest effect, the prevention of dementia is the most effective approach to reducing the disease burden. While multifactorial risk reduction trials have shown promise for dementia prevention^6^ they are often resource-intensive and not easily scalable for widespread implementation at a population level. Consequently, there is an urgent need to develop pragmatic public health options for global risk reduction in dementia, for which effective blood pressure (BP) lowering may be a key strategy.^7 8^ Although there is considerable randomised evidence to support BP lowering reducing the risks of cognitive decline and dementia, these data are derived from older adults with high CV risk, which leaves gaps in knowledge about the appropriateness of such treatment in mid-life despite a strong evidence base linking hypertension in mid-life to poorer cognition and increased dementia risk.^7 9^ Additionally, recent developments in the formulation of combination generic antihypertensive drugs at variable doses provide a potential means for pragmatic, scalable and deliverable risk reduction solutions for dementia.^10 11^

The COVID-19 pandemic challenged face-to-face interactions and limited the opportunity for research, but advanced opportunities for videoconferencing and remote delivery of medical, psychological and psychiatric services. Studies support the validity and reliability of administering videoconference-based neuropsychological tests in rural and urban settings,^12^ and diagnostic outcomes are comparable to traditional face-to-face assessment of neurocognitive disorders.^13^

Herein, we outline the Action To promote brain HEalth iN Adults (ATHENA) study, which was designed to test the feasibility of an online and telehealth clinical trial of BP-lowering as adjunct strategy to standard care for the modification of CV risk to prevent future cognitive decline. Key aims of this study were to assess the feasibility and applicability of recruitment using home BP monitoring, routine blood biochemistry and videoconference measures of cognition, in order to define a population at higher-than-average risk of dementia by virtue of having early subclinical measures of cognitive impairment. A single, fixed low-dose, combination BP-lowering pill (a ‘Triple Pill’ containing telmisartan 20 mg, amlodipine 2.5 mg and indapamide 1.25 mg) was selected as study medication as there is evidence that fixed-dose combination medications provide adequate BP control without adversely affecting the side effect profile. However, since the low-dose Triple Pill has not been tested in older adults at increased risk of cognitive decline, we also aimed to determine the short-term tolerability, safety and adherence to, compared with matching placebo, in participants.

## Methods

### Trial design and oversight

A decentralised, double-blind, placebo-controlled, randomised pilot trial with a low-dose Triple Pill (telmisartan 20 mg, amlodipine 2.5 mg, and indapamide 1.25 mg vs placebo) was undertaken in participants deemed to be at high risk of dementia with moderately raised home BP. The George Institute for Global Health (TGI) coordinated the trial, managed the database, and performed the analyses. Study oversight was conducted by a Steering Committee, comprised of international experts in the fields of dementia, neurology, neuropsychology, cardiology, hypertension, CV disease, public health, geriatric medicine, statistics, epidemiology and clinical trials. This committee was responsible for development and execution of the study design, protocol, data collection and analysis plan as well as drafting reports. The study medications were purchased from regulatory-approved manufacturers, and encapsulated and packaged by PCI pharma services, a Therapeutic Goods Administration current Good Manufacturing Practices licensed manufacturing facility in Melbourne, Australia. The tablets selected were based on the requirement for no more than half an existing dosage form. Each dose of the three study drugs was placed in a capsule; a process that was followed for each tablet and 100% verified by a second manufacturing staff member. The capsules were then filled with microcrystalline cellulose, to prevent rattle, and semiautomatically closed. Matched placebos were packaged in a similar way. This trial was registered with the Australian New Zealand Clinical Trials Registry (ANZCTR).

### Patients, procedures and randomisation

Potential participants were eligible if they were aged between 50 and 70 years and had moderately raised BP (systolic >120 mm Hg and <160 mm Hg or diastolic >80 and <95 mm Hg), and another enrichment factor that indicated elevated risk of cognitive decline. The enrichment factors were through self-report of any of the following: monotherapy treatment of hypertension, diabetes mellitus, elevated low-density lipoprotein cholesterol, obesity, current smoking or a first-degree relative with dementia. Full details of the inclusion and exclusion criteria are outlined in online supplemental table 1.

**Figure 1:**
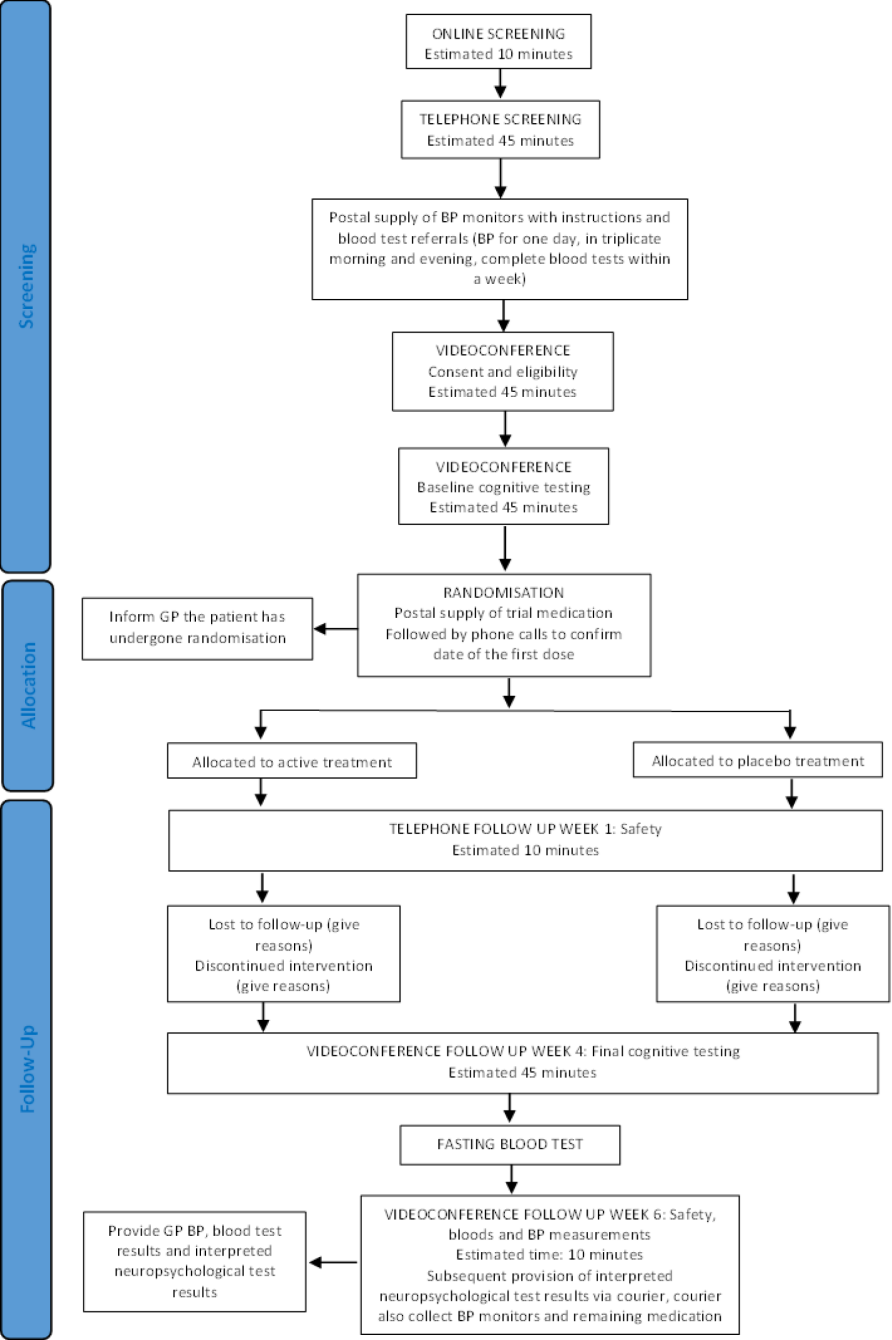
Study procedures. BP, blood pressure; GP, general practitioner.

Participants were recruited through social media (mainly using TrialFacts,^14^ a company that specialises in social media recruitment for clinical trials) and a community campaign led by TGI. The trial was conducted online, without in-person interaction between study personnel and participants. A four-stage screening process was used (figure 1), which consisted of (1) an online questionnaire to assess initial eligibility with questions on enrichment factors including elevated or high cholesterol; (2) telephone consultation for consent, collection of demographic and clinical data and a screening assessment using the Modified Telephone Interview for Cognitive Status,^15 16^ a 13-item telephone-based screening tool to detect pre-existing dementia or significant cognitive impairment; (3) daily home BP monitoring using a certified OMRON device and the collection of fasting bloods for assessment of routine biochemistry (electrolytes and renal function), liver function and lipids and (4) a brief assessment with a neuropsychologist to determine baseline cognitive function using the Test of Premorbid Function, Montreal Cognitive Assessment for Dementia^17^ and an additional five neuropsychological tests to derive a Global Cognitive Composite.^18^,^20^ The tests assessed memory, processing speed and executive function and comprised the Rey Auditory Verbal Learning Test delayed recall (RAVLT- delayed recall), Symbol Digit Modalities Test (SDMT) oral version, Verbal Fluency (letters (F, A, S) and semantic animals), and Oral Trail Making Test. Participants also completed the Cogstate Online Brief Battery to assess psychomotor function, attention, visual learning and working memory, and executive function.^21^ According to the results, a neuropsychologist determined according to normative data, if any test performance was <1 SD below expected limits and in turn, if an individual met standard diagnostic criteria for a minor neurocognitive disorder.

After confirmation of eligibility, participants were randomised according to participant sex via an allocation list generated by an independent statistician to Triple Pill (telmisartan 20 mg, amlodipine 2.5 mg and indapamide 1.25 mg) or a placebo Triple Pill in the form of study capsules, distributed by mail to the homes of participants from a central pharmacy (Syntro Health, Melbourne, Australia). During the randomisation visit, participants were asked to complete the Patient Health Questionnaire (PHQ-9)^22^ and Pittsburgh Sleep Quality Index (PSQI)^23^ via a Redcap online survey, as measures of depressive symptoms and sleep quality, respectively. Participants were required to take their study medication daily for 4 weeks.

Follow-up visit 1 was via a telephone call at 7 days postrandomisation. Subsequent follow-up visits occurred at 4 and 6 weeks through videoconference by trained staff who remained unaware of randomised treatment assignment. BP was self-measured by participants following measurement guidelines of the American Heart Association (AHA).^24^ They were provided with the documentation needed to attend local laboratories for fasting blood tests at the beginning of the study and at 6 weeks of: electrolytes (Na^+^, K^+^), and renal (ie, blood urea nitrogen, serum creatinine, estimated global filtration rate) and liver function (aspartate aminotransferase, alanine transaminase); glucose; and low-density lipoprotein, high-density lipoprotein, total cholesterol and triglycerides.

### Patient and public involvement

Prior to the conduct of this study, The George Institute’s Brain Health Consumer panel were consulted with regards to study design, consent process and other patient facing documents. The panel provided suggestions to improve wording of the patient information and consent form. The Brain Health Consumer panel consists of people with lived experiences, caregivers and representatives of community organisations. The results of this study will be disseminated within the community through media engagement.

### Outcomes

The prespecified primary outcome was *feasibility*, expressed by a percentage with a 95% CI of participants randomised to those screened. That is, the numerator was the number of participants randomised and the denominator was the number of participants who commenced the eligibility questionnaire via the TrialFacts screening form. Reasons for screen failure were recorded, as were reported motivations to participate in the study through an exit survey.

Secondary outcomes were:

1. *applicability* of videoconference assessments, calculated as percentages, with 95% CI for: the number of eligible participants who began videoconference testing (numerator) divided by the number of participants who met eligibility criteria via telephone screening (denominator) and the number of participants who began videoconference testing at 4-week follow-up (numerator) by the number of randomised participants (denominator).
2. *feasibility* of the trial was calculated as a percentage with 95% CI of those that completed all remote assessments (numerator) over number of randomised participants (denominator).
3. *tolerability* of the Triple Pill during 6 weeks of follow-up measured in randomised participants as: the number of prespecified adverse events of special interest (AESI) covering headaches, syncope or collapse, falls, pedal oedema, hypokalaemia, hyperkalaemia and hyponatraemia, recorded in the database; and the number of participants who withdrew from treatment.
4. prespecified *safety* according to all serious adverse events (SAE) that were reported between time of enrolment/randomisation through to trial completion.
5. *adherence* to study medication according to self-reported pill count of missed doses.

Other feasibility outcomes are outlined below.

1. Administration of neuropsychological assessments via videoconference, calculated as the percentage of participants who completed neuropsychological assessments, with reasons for failure collected.
2. Self-completion of online Cogstate brief battery assessed by completion rates, calculated as the number of participants who completed the assessment (numerator) over the number who began the assessment (denominator), at screening and at week 4. Cogstate test performance criteria were applied to determine whether sufficient responses were recorded to allow computation of a reliable outcome measure to categorise whether tests were passed, a performance or completion failure. Where a participant had a completion failure in any test, they were deemed as not completing the assessment.
3. Applicability of BP monitoring at home, calculated as the number who completed at home BP self-measurement (numerator) over the number eligible from telephone screening (denominator).

We also assessed the size of the treatment effect as preintervention and postintervention change with 95% CI on the composite and individual component scores for memory, processing speed and executive function in the global cognitive composite and Cogstate results. Effect size changes for total PHQ-9 and PSQI scores were measured preintervention and postintervention, as were changes in BP (systolic and diastolic).

### Post-trial survey

All participants who were randomised or completed stage 3 of the screening process (at home testing) were invited to participate a post-trial survey (online supplemental table 2) to assess their satisfaction with the procedures and outline motivating factors for their participation. For randomised participants, survey results were linked to their participant identification number. For all participants, consent to the survey was implied in those who completed the survey. All responses were recorded anonymously in the database.

### Sample size

As ATHENA is a pilot feasibility study to inform the sample size calculation of a large-scale trial, we aimed to include 50 participants to be randomised, based on recommendations by Sim & Lewis.^25^ For feasibility and pilot trials, the sample size per arm ranges from 10 to 300 participants (median 36, IQR 25–50) for trials with dichotomous endpoints. To achieve the recruitment target of 50 participants in our trial, 200 potential participants were estimated to require video teleconference neuropsychological tests, 650 for self-home BP monitoring and 2000 for online screening.

### Statistical analysis

At each stage of the study screening process, the number of participants screened and subsequently eligible at each stage were recorded. Feasibility and applicability outcomes were expressed as percentages with 95% CI, constructed using the standard formula for a binomial CI. Baseline characteristics were summarised according to treatment allocation. Test scores (Preclinical Alzheimer’s Cognitive Composite 5 [PACC5]: RAVLT delayed recall, SDMT, Oral Trials B, Phonological Fluency, Semantic Fluency, PHQ-9 total, PSQI Global, Cogstate (Detection, Identification, One Card Learning, One Back, Groton Maze Test) were summarised using mean (SD) preintervention and postintervention, with differences calculated with paired t tests. Ladder plots were used to graphically display test scores for each randomised participant. Changes in BP from baseline were calculated as mean difference with 95% CI by randomised treatment. The overall BP difference between Triple Pill and placebo was calculated using a linear mixed effects model, with postrandomisation BP as the dependent variable, fixed effects for baseline BP, treatment group, visit (week), and an interaction between the treatment variable and visit and patient as a random effect. BP variability was calculated for each participant as the SD of their BP measures from weeks 1–4 and summarised as means according to treatment allocation.

Exit survey data were summarised visually. Bar charts were used to present the answers to nine questions for participant motivation for entry in the study. Cronbach’s alpha was used to measure the internal consistency of the motivation questions calculated via pairwise correlations between questions in the survey. Answers to two free-text questions eliciting information on participant’s motivation to enter the trial, and suggestions to motivate others to participate in a similar trial, were summarised using word clouds derived using the wordcloud package in R. All analyses were undertaken R V.4.2.0.^26^

### Data availability statement

Deidentified unpublished data on individual test scores for PACC5, RAVLT Delayed recall, SDMT, Oral Trials B, Phonological Fluency, Semantic Fluency, PHQ-9 total, PSQI Global and Cogstate can be made available on reasonable request to the corresponding author.

## Results

### Feasibility and applicability of the trial

**Figure 2:**
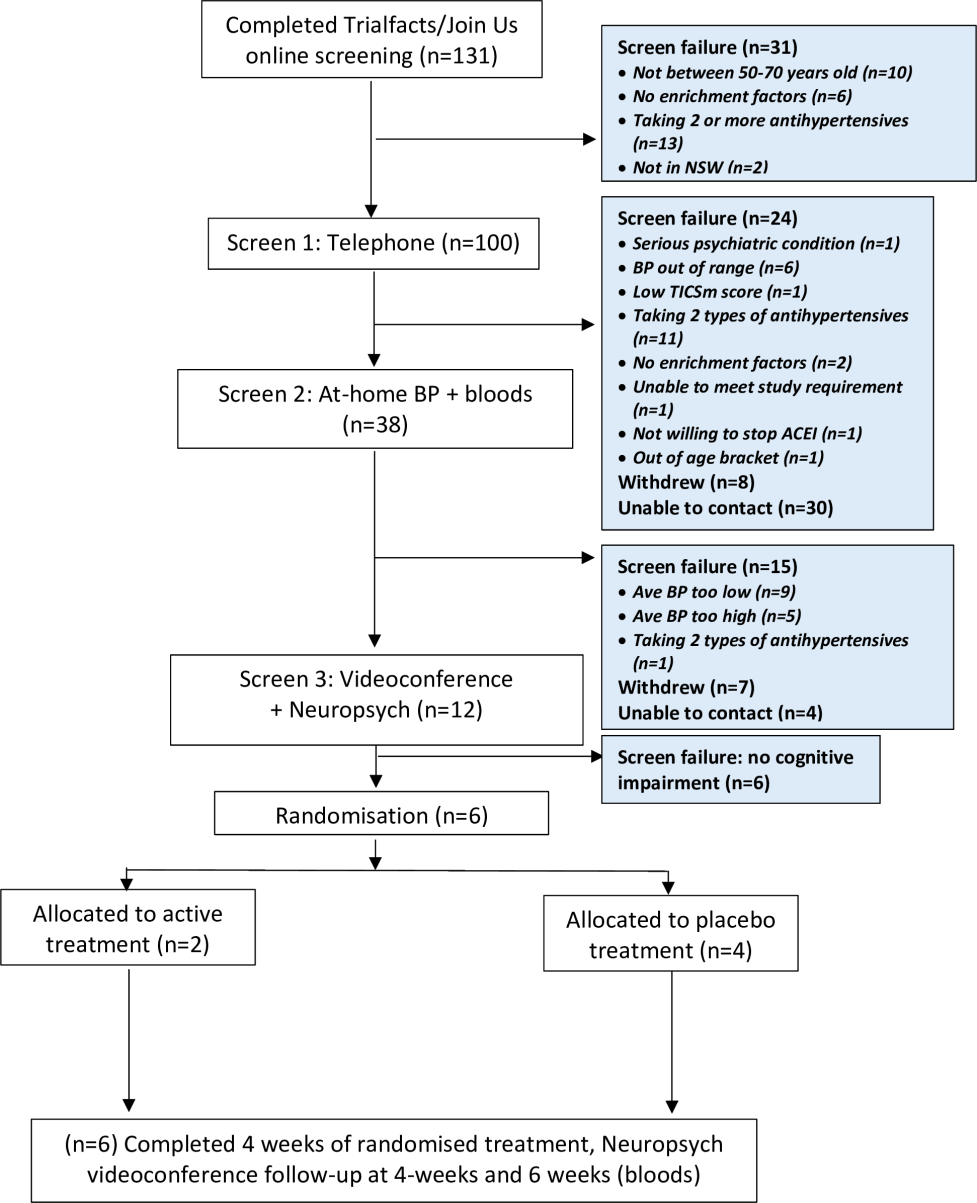
Recruitment steps. BP, blood pressure; TICSm, Modified Telephone Interview for Cognitive Status; NSW, New South Wales; ACEI, angiotensin converting enzyme inhibitor.

The ATHENA study recruited from 12 October 2021 to 3 February 2022. A total of 131 participants completed the online screening, of whom 100 were eligible for telephone screening (figure 2). There were 31 participants who failed online screening; the main reasons were already taking ≥2 antihypertensives (n=13) and being outside the eligible age range (n=10). Another 62 participants were failures at telephone screening (see figure 2 for detailed reasons). This left 38 eligible people for home BP and blood testing, in whom 12 were able to proceed to screening stage 3 (videoconference with neuropsychological testing) and 6 were randomised into the study.

The feasibility of the study, as assessed by the proportion of patients randomised out of those screened, was 5% (6/131; 95% CI 2 to 10%) (table 1). Applicability of the assessments administered via telehealth at screening was 32% (95% CI 18% to 49%). There were two randomised participants who were unable to complete all remote assessments, one failed to complete all neuropsychological testing (lost connection) and one failed to monitor or record their BP/heart rate from weeks 1–6. Thus, the overall applicability of the trial was 67% (95% CI 22% to 96%).

**Table 1:** Primary, secondary and tertiary outcomes of the ATHENA study

Outcome	n	N	% (95% CI)
Primary			
Feasibility of study (proportion of participants to those screened)	6	131	5 (2 to10)
Secondary			
1. Applicability of videoconference assessments			
(i) Eligible participants who begun videoconference testing after meeting eligibility criteria via telephone screening	12	38	32 (18 to 49)
(ii) Participants who begin videoconference testing at 4 weeks follow-up after being randomised	6	6	100 (54 to 100)
2. Applicability of the trial	4	6	67 (22 to 96)
3. Tolerability during follow-up at 6 weeks:			
a. Adverse events of special interest	2	6	
b. Participant withdrawal from treatment	0	6	
4. Serious adverse event	0	6	
Tertiary			
1. Feasibility of videoconference neuropsychological assessments	12	12	100 (74 to 100)
2. Feasibility of completing CogState brief battery independently online			
At screening	13	16	81 (54 to 96)
At week 4	5	6	83 (36 to 100)
3. Applicability of BP monitoring at home.	27	38	71 (54 to 85)

**Primary**—feasibility of the study: the denominator is the number of participants who commence the eligibility questionnaire via the TrialFacts screening form, and the numerator is the number of participants randomised.

**Secondary**—applicability of videoconference assessments: (i) the number of eligible participants that begin video conference testing (numerator), divided by the number of participants meeting eligibility criteria via telephone screening (denominator); (ii) the number of participants who begin videoconference testing at 4-week follow-up (numerator) over the number of randomised participants (denominator). (1) Applicability of the trial—The number of participants that complete all remote assessments (numerator) over number of randomised participants (denominator), (2) Tolerability during follow-up at 6 weeks: (a) Adverse Events of Special Interest (pre-specified options in the database were: headache, syncope/collapse, falls, pedal oedema/ankle swelling, hyperkalaemia, hypokalaemia, hyponatraemia). (b) Participant withdrawal from treatment. (3) Safety: any serious adverse event (SAE).

**Tertiary**: (1) Feasibility of completing the videoconference neuropsychological assessments—percentage of participants who complete videoconference neuropsychological assessments once testing has begun, (2) Feasibility of completing CogState brief battery independently online—the number of participants that complete the assessment (numerator) over the number that begin the assessment (denominator). (Note: Calculated at screening and at week 4), (3) Applicability of BP monitoring at home—The number that completed home BP self-measurement (numerator) over the number eligible from telephone screening (denominator). Remaining tertiary outcomes are effect size changes and presented in online supplemental tables.

ATHENAAction To promote brain HEalth iN AdultsBPblood pressure

All the 12 (100%) participants who began the videoconference neuropsychological assessments were able to complete them. Feasibility of completing the online independent Cogstate brief battery was 81% (95% CI 54% to 96%) at screening, since 13 out of 16 completed the assessment. In those randomised, six began the Cogstate assessment and five completed it, yielding a feasibility of 83% (95% CI 36% to 100%).

Of the 38 participants who entered the home BP self-measurement, 27 were able to complete all assessments; thus, applicability of home BP monitoring was 71% (95% CI 54% to 85%).

### Participant characteristics and outcomes

Baseline characteristics of the six randomised participants (three female) are presented in table 2. Two participants were allocated to active treatment and four to placebo. Median (range) age at baseline was 61.7 years (IQR 56.5–70.5), and mean (SD) systolic BP were 128.8 mm Hg (7.4) and 138.9 mm Hg (1.6) in the placebo and active treatment arms, respectively. In terms of comorbidities at screening, one person had a previous stroke, one person had a fall in the last 12 months, four had hypertension and five had elevated cholesterol. Three participants confirmed they had a parent with dementia.

**Table 2:** Participant baseline characteristics by randomised treatment

	Placebo	Triple Pill	Overall	Overall
	Mean (SD)	Mean (SD)	Mean (SD)	Median (range)
Number of participants	4	2	6	6
Female	2	1	3	3
Age, years	62.7 (6.4)	63.5 (9.9)	63.0 (6.7)	61.7 (56.5, 70.5)
Height, cm	166.5 (10.0)	165.0 (9.9)	166.0 (8.9)	168.5 (153.0, 175.0)
Weight, kg	89.5 (13.2)	66.5 (6.4)	81.8 (15.9)	80.5 (62.0, 105.0)
BMI	32.5 (5.5)	24.4 (0.6)	29.8 (6.0)	28.11 (24.0, 39.3)
Smoking				
Never smoker	2	1	3	3
Ex-smoker	2	1	3	3
Alcohol, >1 per week	2	2	4	4
Highest level of education				
Undergraduate degree	1	1	2	2
Postgraduate degree	2	1	3	3
Other	1		1	1
Systolic BP	128.8 (7.4)	138.9 (1.6)	132.2 (7.8)	133.3 (121.7, 140.1)
Diastolic BP	83.1 (2.1)	87.9 (4.1)	84.7 (3.5)	84.4 (80.0, 90.8)
Heart rate	72.6 (3.2)	68.2 (NA)	71.7 (3.4)	72.7 (68.2, 76.2)
Glucose, mmol/L	5.3 (0.45)	5.0 (0.28)	5.2 (0.40)	5.2 (4.7, 5.7)
Total cholesterol, mmol/L	4.7 (1.04)	5.6 (0.57)	5.0 (0.97)	5.2 (3.2, 6.0)
LDL, mmol/L	2.75 (0.91)	2.90 (0.14)	2.8 (0.71)	2.9 (1.5, 3.6)
HDL, mmol/L	1.23 (0.34)	2.25 (0.35)	1.57 (0.61)	1.45 (0.90, 2.50)
Triglycerides, mmol/L	1.55 (0.40)	0.95 (0.21)	1.35 (0.45)	1.30 (0.80, 1.90)
Creatinine, µmmol/L	87.5 (22.5)	80.0 (14.1)	85.0 (19.0)	90.0 (55.0, 105.0)
Sodium, mmol/L	139.0 (2.9)	138.5 (2.1)	138.8 (2.5)	139.5 (135.0, 142.0)
Potassium, mmol/L	4.35 (0.39)	4.40 (0)	4.37 (0.30)	4.35 (4.00, 4.90)
BUN /urea, mmol/L	5.05 (1.41)	6.00 (0.99)	5.37 (1.27)	5.7 (3.6, 6.7)
eGRF, mL/min/1.73m^2^	70.3 (13.3)	81.5 (3.5)	74 (11.9)	72 (61, 90)
AST, IU/I	21.8 (5.5)	35.0 (1.4)	26.2 (8.1)	26 (17, 36)
ALT, IU/l	30.8 (14.8)	21 (0)	27.5 (12.5)	21.5 (16, 49)

ALTalanine transaminaseASTaspartate aminotransferaseBMIbody mass indexBPblood pressureBUNblood urea nitrogeneGFRestimated glomerular filtration rateHDLhigh-density lipoproteinLDLlow-density lipoprotein

In presenting all planned outcomes according to standards of reporting of clinical trial data, we acknowledge extreme imprecision in the estimates of the treatment effect due to the very small sample size, such that these results should be interpreted cautiously. Effect size changes of the cognitive assessments are presented in online supplemental table 3. Changes in test scores preintervention and postintervention are presented as ladder plots: PACC5 total and mean (online supplemental figure 1), RAVLT delayed recall, SDMT, Oral Trials B, Phonological Fluency, Semantic Fluency (online supplemental figure 2), and PHQ-9 total, PSQI Global (online supplemental figure 3). There were no consistent results seen between groups in the cognitive assessments.

The effect of the Triple Pill (vs placebo) for systolic and diastolic BP over 4 weeks was −8.4 mm Hg (95% CI −79.6 to 62.9) and −14.8 mm Hg (95% CI −43.1 to 13.5), respectively. The mean systolic BP variability for participants on the Triple Pill was 16.4 mm Hg compared with 10.2 mm Hg for those on placebo.

In the six randomised participants, two AESIs were reported, one headache and one hyperkalaemia (K^+^ 5.5); there were no SAEs or withdrawals from treatment. In terms of medication adherence, one participant reported one dose missed in weeks 1–4. At the week 6 follow-up assessment, five participants reported no study medication remaining, and one reported only two capsules were remaining.

### Post-trial survey

**Figure 3:**
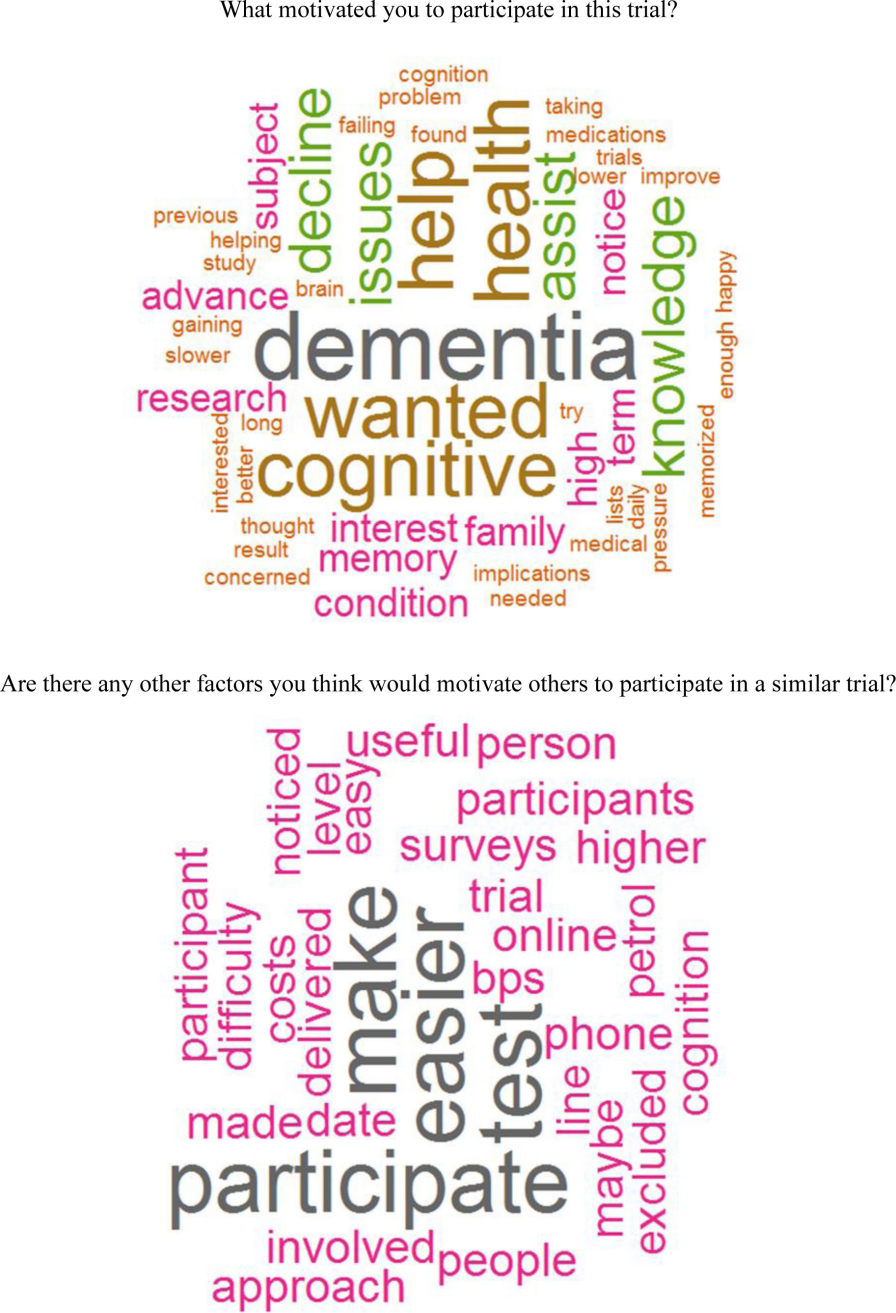
Word clouds from exit survey.

The exit survey was completed by 21 participants (4 randomised and 17 screened but not randomised). They all stated that the procedures were easy to follow. Participants were motivated to participate in the trial because they were concerned about dementia, cognition and their health (figure 3). This was reflected in the answers given in the prespecified motivation questions (online supplemental figure 4) suggesting that being at increased risk from subjective memory issues was ‘fairly’ or ‘very’ important (16/21) as was getting access to the treatment if proven to be safe and effective after the trial (17/21). Receiving a gift card was not an important reason for participation (11/21). Internal consistency of the motivation questions was good (Cronbach’s α=0.854, 95% CI 0.62 to 0.91).

## Discussion

The primary goal of our study was to evaluate different aspects of the feasibility of using social media and online screening with remote neurocognitive and home BP assessments as methods for conducting a decentralised clinical trial in participants at increased risk of dementia. The main finding is that the four stages of remote screening and recruitment of older adults with increased risk is not readily feasible for a large clinical trial of the prevention of dementia.

Of 131 expressions of interest through social media, there were 125 screen failures, which may reflect the non-targeted non-personal approach to participation (49 participants withdrew or did not answer when contacted) or use of an overly arduous, screening process. Recruitment of participants in the ATHENA study were through social media via TrialFacts and social media accounts X (formerly Twitter) and Facebook. In addition, we performed a community campaign through a clinical trial registry and by trial advertisement in primary care physician clinics. A majority of potential participants came through social media suggesting that while this type of recruitment campaign may initially capture the attention of the target population, it does not necessarily translate to commitment to participate in the trial as evidenced by 30 non-contactable and 8 withdrawals of potential participants after completing online screening (figure 1). Our results do not clearly indicate a preferential approach to recruitment, they may also suggest that a broad range of recruitment techniques are required for such trials of prevention. However, the study did show that among eligible participants, the conduct of online videoconference assessments (baseline and follow-up neuropsychological assessments) and self-completion of an online cognitive test was feasible and applicable. An important advantage of this approach is the wide reach and inclusivity, and opportunity to avoid significant costs and constraints of time and travel for participants and research staff.^27^ Being able to undertake research procedures at home or another familiar environment can alleviate anxiety, as noted during COVID-19 restrictions.^28^ However, some of the reported pitfalls in remote trial delivery include a lack of connectedness, which may impact on retention and potential bias towards those who are computer literate or have access to the internet.^27^

As populations continue to age, dementia is rapidly increasing public health concern. Prevention is strongly advocated by the WHO,^29^ AHA^30^ and other leading organisations, where the ultimate success of defining approaches will depend on several factors, one of which being the ability to recruit the necessary large number of participants for clinical trials to test applicable strategies.^31^ There are many challenges to the inclusion of older people in clinical trials ^31 32^: methods of obtaining informed consent, access issues, ethnic barriers, subject fears and concerns, gatekeeper influence, comorbidity and competing risks of mortality. Proposed solutions to incorporating optimal participant recruiting methods include the tailoring of processes to suit the target population, such as: telephone calls for home-bound populations, radio or newspaper advertisements in participants aged ≥65 years; and the use of mailings and fliers about research. Other solutions include the removal of access barriers through the provision of remote trial delivery options or defraying the costs of transportation, and the provision of clear communication to potential participants and their caregivers.^32^

Hypertension is a key modifiable risk factor dementia and cognitive decline.^7 33^ The proposed mechanisms include the contribution of elevated BP to cerebrovascular disease, endothelial cell dysfunction, damage to microvessels, subclinical cerebral infarction, cerebral white matter lesions, inflammation and the modification of Alzheimer’s disease pathology.^34^ However, questions remain as to the role of BP lowering for the protection of cognitive function. Clinical trials, such as Systolic Blood Pressure Intervention Trial-Memory and Cognition in Decreased Hypertension, which showed that a reduction in mean systolic BP from 139.7 mm Hg to 121.6 mm Hg was associated with a 15% reduced risk of incident dementia and mild cognitive impairment,^35^ have not been designed primarily for the prevention of dementia.^36^ There is a clear need of a risk reduction trial that to reliably determine the role of BP lowering for the prevention of dementia.

There are several limitations to our study. As a small feasibility study, it was inevitably unable to provide precise estimates, but the focus was on feasibility and applicability of certain trial procedures. Another limitation was that it was conducted in only one state of Australia, and we cannot be sure that the findings would be similar in a wider context.

We recommend a simplified one to two stage screening process to improve the recruitment of a trial using antihypertensive medications to attenuate cognitive decline in high-risk adults. Codeveloping recruitment strategies with people with lived experience of cognitive decline and their caregivers likely will improve feasibility.

In conclusion, we report the feasibility of administering a decentralised clinical trial conducted via telehealth to assess the applicability of online recruiting and remotely assessing a population of people who likely had early stages of declining memory and higher-than-average risk of dementia. While studies with multiple screening processes in a non-targeted population are not feasible, the use of home cognitive and other assessments are applicable to those who meet eligibility criteria.

## supplementary material

10.1136/bmjopen-2023-080862 online supplemental file 1

## Data Availability

Data are available upon reasonable request.
