# Supplementary material for: Randomised controlled decentralised feasibility trial of a fixed low-dose combination antihypertensive drug strategy to attenuate cognitive decline in high-risk adults
Source: BMJ Open. 2024 Aug 24;14(8):e080862. doi: 10.1136/bmjopen-2023-080862 (PMC11344491; doi:10.1136/bmjopen-2023-080862)
Supplement: online supplemental file 1 [file bmjopen-14-8-s001.pdf]

Supplementary table of contents:

1. **Supplementary Table 1: Full inclusion and exclusion criteria**
2. **Supplementary Table 2: Post-study survey**
3. **Supplementary Table 3: Effect size changes of cognitive assessments**
4. **Supplementary Figure 1: Ladder plot of neuropsychological composite score**
5. **Supplementary Figure 2: Ladder plot of individual component scores of Preclinical Alzheimer Cognitive Composite (PACC5) (RAVLT delayed recall, SDMT, Oral Trials B, Phonological Fluency, Semantic Fluency)**
6. **Supplementary Figure 3: Ladder plot of PHQ9 Total, PSQI Global, pre and post intervention**
7. **Supplementary Figure 4: Prespecified motivation questions from exit survey**
8. **Action To promote brain HEalth iN Adults (ATHENA) protocol**

Supplementary Table 1: Full inclusion and exclusion criteria

Inclusion Criteria:

- Age 50 to 70 years.
- Moderately raised BP (SBP >120 and <160 mmHg or DBP > 80 and < 95 mmHg), whether or not they are on any treatment, or on treatment with a single agent at low to moderate dose.
- DSM-V diagnosis of Minor Neurocognitive Disorder:
    - modest cognitive decline from a previous level of performance in at least one domain, based on the concerns of the individual, a knowledgeable informant or the clinician; and a decline in neurocognitive performance of >1 standard deviation below appropriate norms on formal testing or equivalent clinical evaluation.
    - cognitive deficits are insufficient to interfere with daily activities, but that greater effort, compensatory strategies, or accommodation may be required to maintain independence.
    - cognitive deficits do not occur exclusively in the context of a delirium.
    - cognitive deficits are not primarily attributable to another mental disorder (for example major depressive disorder and schizophrenia).
- An additional enrichment factor indicating elevated risk for declining cognition, defined as one or more of self-reported: monotherapy treatment of hypertension, diabetes mellitus, elevated low-density lipoprotein cholesterol, obesity, current smoking, or first degree relative with dementia.
- No clear contraindication to any of the study treatments.
- Provision of online, verbal and electronic informed consent.

Exclusion Criteria:

- Taking an ACE-I that cannot be:
    - stopped, or
    - switched to open label telmisartan 20-40mg, indapamide 1.25mg, or amlodipine 2.5-5mg, or
    - switched to a beta blocker
- Contraindication to any of the study medications, in the context of BP lowering medication currently prescribed by primary care physicians (e.g. those who are on regular NSAID prescription/consumption).
- Unable to complete the study procedures and/or follow-up.
- Significant abnormal kalaemia and/or natraemia, in the opinion of the responsible physician.
- Stage 3b renal failure (GFR < 45 ml/min/1.73m^2).
- Severe liver disease (e.g. acute viral hepatitis, chronic active hepatitis, cirrhosis).
- Severe hepatic impairment (ALT or AST) >3x the upper limit of normal [ULN].
- Pre-existing dementia, another neurodegenerative disease (e.g. Huntington's, multiple sclerosis, Parkinson's disease), cognitive decline due to substance use (measured on the World Health Organisation Alcohol Use Disorders Identification Test (WHO-AUDIT)), severe mental ill-health, or neurological or systemic disorder.
- History of stroke within the last 6 months and/or history of stroke with any residual deficit.
- History of traumatic brain injury with loss of consciousness within the last 2 years.
- No ongoing serious medical or psychiatric condition that would prevent full participation.

Supplementary Table 2: Post-study survey

**Q1.0 What motivated you to participate in this trial?**						
**Q2. Did you find the procedures easy to follow? ☐ Yes ☐ No If no, what did you like or not like.**						
**3. Using a scale of 1 = Not at all important to 5 = Very important, please rate the following factors that you think are important to motivate others to join a similar trial?**						
	**Not at all Important 1**	**Slightly important 2**	**Important 3**	**Fairly important 4**	**Very important 5**	**No opinion**
**a) Being at increased risk because of family history of dementia**	☐	☐	☐	☐	☐	☐
**b) Being at increased risk because you personally are starting to have memory issues**	☐	☐	☐	☐	☐	☐
**c) Getting access to support during the trial – information, brain training apps and tips etc**	☐	☐	☐	☐	☐	☐
**d) Getting results of brain scans and blood tests**	☐	☐	☐	☐	☐	☐
**e) Getting access to the treatment, if proven safe and effective, after the trial**	☐	☐	☐	☐	☐	☐
**f) Making a contribution to research**	☐	☐	☐	☐	☐	☐
**g) Personal interest**	☐	☐	☐	☐	☐	☐
**h) Receiving a copy of results**	☐	☐	☐	☐	☐	☐
**i) Receiving a gift card**	☐	☐	☐	☐	☐	☐
**j) Other**<br>**Specify ___________________**	☐	☐	☐	☐	☐	☐

Supplementary Table 3: Effect size changes of cognitive assessments

Randomised Participants overall			
**Measure**	**Pre**	**Post (4 weeks)**	**Difference Post-Pre**
	Mean (SD)	*Mean (SD)*	*Difference (95%CI)*
PACC5			
Total	-2.48 (3.10)	-1.27 (2.28)	1.21 (-2.59, 5.01)
Mean	-0.50 (0.62)	-0.25 (0.46)	0.24 (-0.52, 1.00)
Neuropsychological tests			
RAVLT delayed recall	0.05 (1.00)	0.46 (1.11)	0.41 (-0.16, 0.97)
SDMT	-1.99 (1.90)	-2.42 (1.70)	-0.43 (-2.19, 1.33)
Oral Trails B	-0.78 (2.08)	0.30 (0.97)	1.08 (-0.83, 3.00)
Phonological Fluency	-0.21 (1.30)	0.19 (1.18)	0.41 (-0.02, 0.83)
Semantic fluency	0.46 (1.16)	0.20 (0.55)	-0.26 (-1.69, 1.17)
PHQ-9			
Q2 Feeling down, depressed, or hopeless	0.50 (0.84)	0.67 (0.52)	0.17 (-0.62, 0.96)
Total	4.83 (3.37)	5.67 (3.56)	0.83 (-3.12, 4.78)
PSQI			
Global	5.33 (0.82)	6.83 (1.17)	1.50 (0.40, 2.60)
Cogstate primary outcome measures			
Detection	2.61 (0.07)	2.61 (0.09)	0.00 (-0.11, 0.10)
Identification	2.80 (0.06)	2.81 (0.05)	0.01 (-0.06, 0.08)
One Card Learning	1.04 (0.12)	1.04 (0.09)	0.00 (-0.14, 0.16)
One Back	1.33 (0.26)	1.25 (0.19)	-0.08 (-0.40, 0.24)
Groton Maze Learning Test	48 (27)	32 (17)	-16 (-56, 25)

Footnote:

Cogstate primary outcome measures description:

- Detection = Reaction time (Log10 milliseconds) Speed of performance; mean of the log10 transformed reaction times for correct responses. Lower score = better performance
- Identification = Reaction time (Log10 milliseconds) Speed of performance; mean of the log10 transformed reaction times for correct responses. Lower score = better performance
- One card learning = Accuracy (Arcsine square root proportion correct) Accuracy of performance; arcsine transformation of the square root of the proportion of correct responses. Higher score = better performance
- One back = = Accuracy (Arcsine square root proportion correct) Accuracy of performance; arcsine transformation of the square root of the proportion of correct responses. Higher score = better performance
- Groton Maze Leaning Test (Total) = Total Errors (Count (number up to 3 digits) Total number of errors made when learning the same hidden pathway across the consecutive learning trials. Lower score = better performance

Supplementary Figure 1: Ladder plot of neuropsychological composite score using the Preclinical Alzheimer Cognitive Composite (PACC5) Total and Mean Score

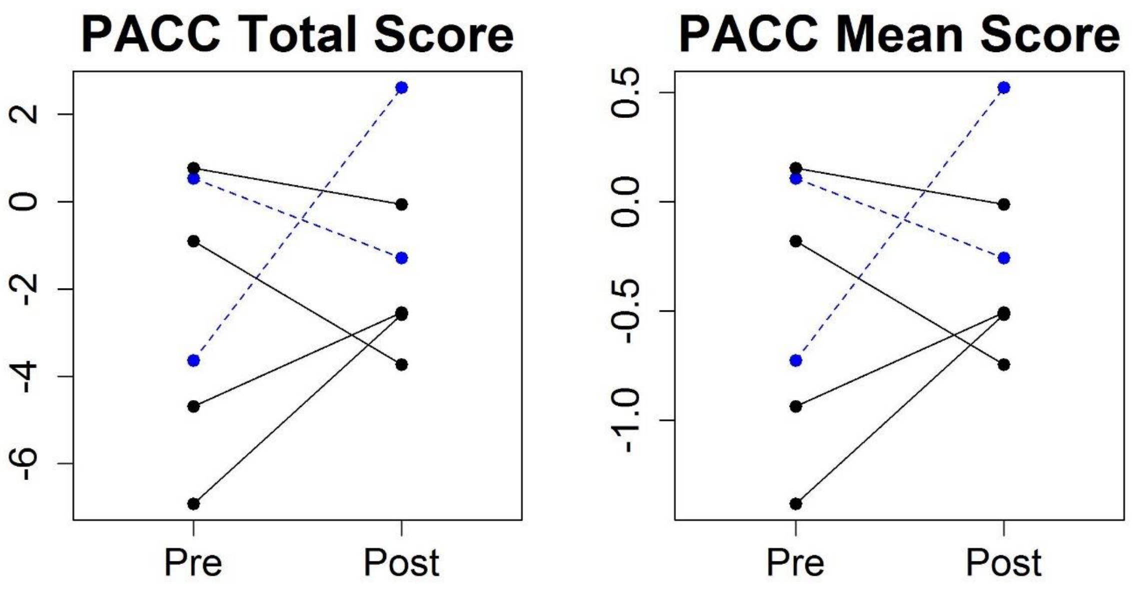

Dashed blue line represents participants on triple pill, solid black line on placebo

Supplementary Figure 2: Ladder plot of individual component scores of Preclinical Alzheimer Cognitive Composite (PACC5) (RAVLT Delayed, SDMT, Oral Trials B, Phonological Fluency, Semantic Fluency)

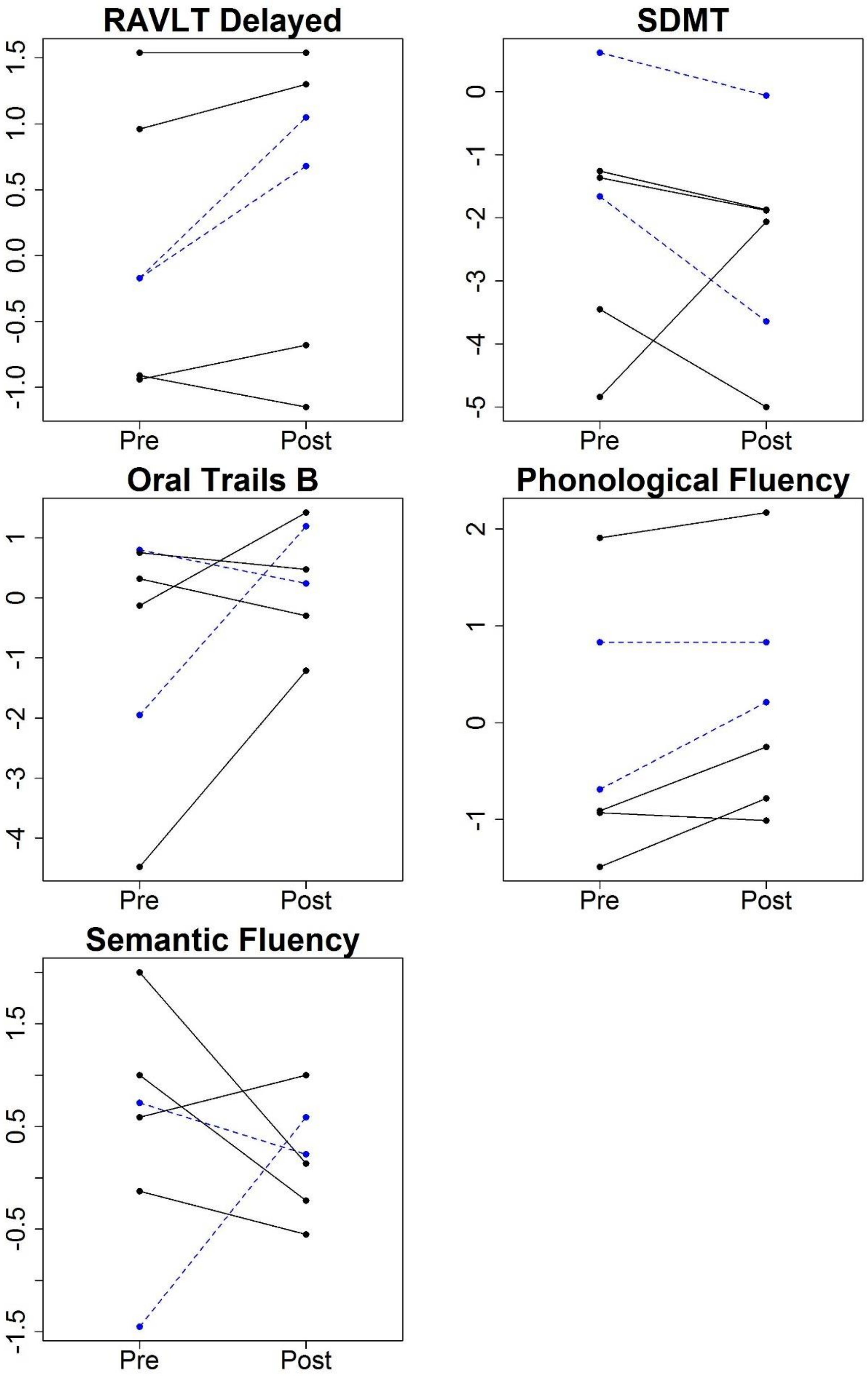

Dashed blue line represents participants on triple pill, solid black line on placebo

Supplementary Figure 3: Ladder plot of PHQ9 Total, PSQI Global, pre and post intervention

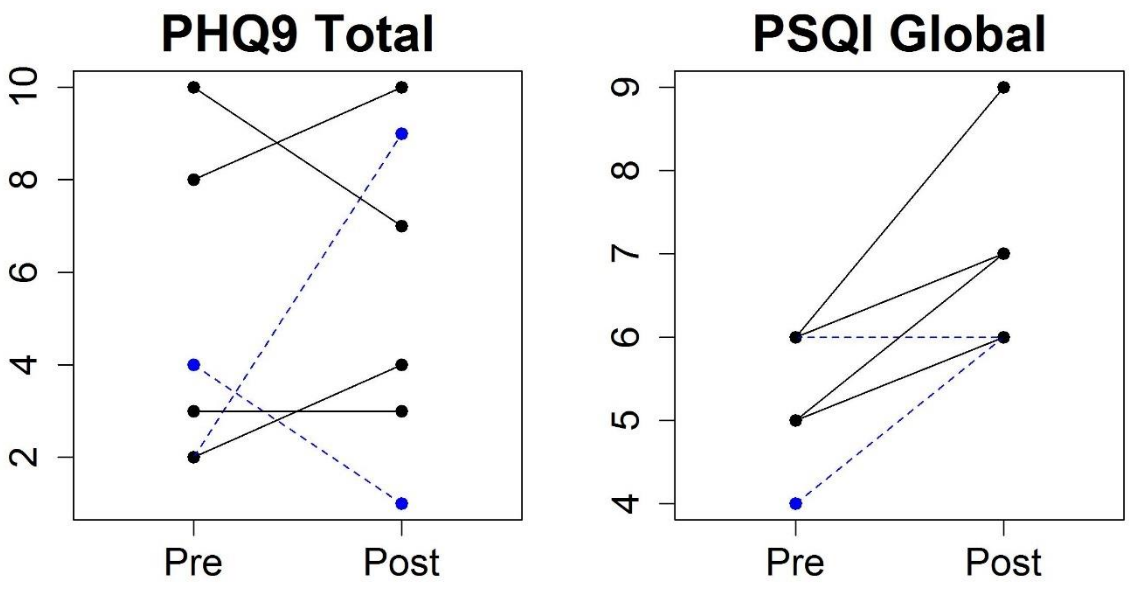

Dashed blue line represents participants on triple pill, solid black line on placebo

Supplementary Figure 4: Prespecified motivation questions from exit survey

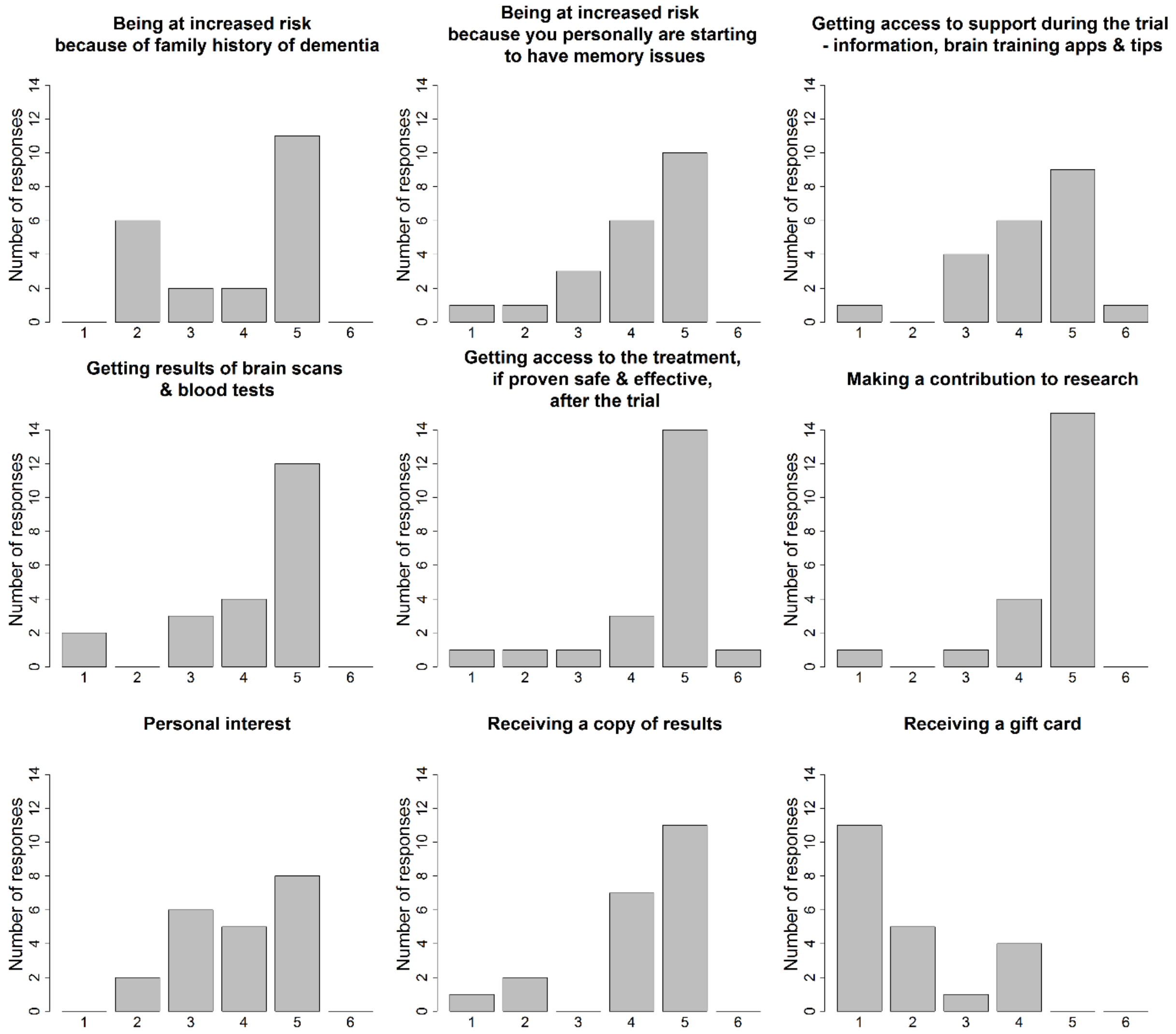

Footnote:

Where response: 1= Not at all important, 2= slightly important, 3= Important, 4= Fairly Important, 5= Very Important, 6= No opinion
