# Supplementary material for: Randomised controlled decentralised feasibility trial of a fixed low-dose combination antihypertensive drug strategy to attenuate cognitive decline in high-risk adults
Source: BMJ Open. 2024 Aug 24;14(8):e080862. doi: 10.1136/bmjopen-2023-080862 (PMC11344491; doi:10.1136/bmjopen-2023-080862)
Supplement: Supplementary file 2 [file bmjopen-2023-080862.reviewer_comments.pdf]

PEER REVIEW HISTORY

BMJ Open publishes all reviews undertaken for accepted manuscripts. Reviewers are asked to complete a checklist review form and are provided with free text boxes to elaborate on their assessment. These free text comments are reproduced below.

ARTICLE DETAILS

**TITLE (PROVISIONAL)**	A randomised controlled decentralised feasibility trial of a fixed low-dose combination antihypertensive drug strategy to attenuate cognitive decline in high-risk adults
**AUTHORS**	Carcel, Cheryl; Clancy, Lauren; Harris, Katie; Peters, Ruth; Byrne, Aisling; Bassett, Kimberley; Freed, Ruth; Hoyos, C. M.; Rodgers, Anthony; Lindley, Richard; Chalmers, John; Xu, Ying; Woodward, Mark; Ouyang, Menglu; Naismith, Sharon; Anderson, Craig

VERSION 1 - REVIEW

**REVIEWER NAME**	***Genevieve Steiner***
**REVIEWER AFFILIATION**	Western Sydney University, NICM
**REVIEWER CONFLICT OF INTEREST**	None
**DATE REVIEW RETURNED**	07-Nov-2023

**GENERAL COMMENTS**	Thank you for the opportunity to review this manuscript. This is a well-reported clinical trial and it is unfortunate that the study protocol was not feasible in this population, particularly because of the substantial evidence-practice gap in dementia prevention studies. The outcome of my review is detailed below.<br><br>For the self-reported enrichment factors that indicated elevated risk of cognitive decline, how was the self-report information captured? Verbally? Or was written evidence required? E.g., verbal self-report of elevated LDL cholesterol seems unlikely.<br><br>Which of the recruitment techniques was the most effective? Some information here would be helpful for other researchers looking to recruit similar populations.<br><br>For participants already receiving monotherapy treatment for hypertension, were there any medication changes in order to participate in the trial? Or did treatment as usual continue?<br><br>RAVLT-D in the methods should be spelled out as RAVLT delayed recall.<br><br>What safety criteria were used for AEs/SAEs and what framework was used for categorisation (severity, type)? There is detail in the supplementary materials, but a little more info needs to be included in the MS.<br><br>What did trial participants do at the end of the trial with any remaining medication? In the full protocol, it looks like it was returned to the pharmacy? Were participants compliant with this? Does this also mean that the medication was dispensed via

	participants' local pharmacies? The success of these methods would help to inform future studies.<br><br>For the linear mixed effects model, which factors were fixed and which were random?<br><br>It would be worth noting the expected sample size in the main body of the manuscript and noting that the full justification is in the supplementary materials.<br><br>Trial registration # should be detailed in the MS. Was the protocol published?<br><br>How specific are the 49/125 screen failures associated with withdrawals or being non-contactable due to the recruitment methods used? The high level of screen failures at this point in the recruitment pipeline suggests that feasibility of the recruitment methods was low. Can the authors provide some additional discussion here please?<br><br>In order to determine whether cognitive deficits interfere with ADLs, how were ADLs measured?<br><br>Given the lack of feasibility, the discussion could benefit a little more depth on what the researchers would do differently next time. Perhaps a 'lessons learned' or 'recommendations' section would be helpful to consolidate the key points (e.g., less screening? Different recruitment methods?).<br><br>Minor:<br>Typos:<br>Page 8, Line 10 – 'lips' should this be lipids?<br>Page 8, Line 50 – Typo should be "complete the Patient…"<br>Page 9, Line 42 – Typo should be "began"<br>Supplementary materials, last line of exclusion criteria – Typo should be "psychiatric condition"<br><br>Figure 2 has some slight formatting issues with arrows now being consistently aligned.

**REVIEWER NAME**	***Takeshi Fujiwara***
**REVIEWER AFFILIATION**	Jichi Medical University
**REVIEWER CONFLICT OF INTEREST**	Nothing
**DATE REVIEW RETURNED**	08-Nov-2023

**GENERAL COMMENTS**	Summary<br>The aim of this randomized controlled trial in adults at high-risk of dementia (totally 131 participants were screened) was to assess the feasibility (primary outcome) and applicability of recruitment using home blood pressure (BP) monitoring, routine blood biochemistry and videoconference measures of cognition in order to define a population at higher-than-average risk of dementia. The feasibility of this decentralized trial was low (5%), but the applicability of remote assessments of cognitive function was acceptable (67%).<br><br>Comments

	・This study had so many variables to assess that it was difficult to understand the results. If possible, it may be possible to narrow down the evaluation variables in this paper and write other evaluation variables in separate papers.<br><br>・How was the sample size calculated before the study started? The small sample size made interpretation of the results difficult.<br><br>・In the introduction section, the authors should clearly state why there was necessary to evaluate the tolerability, safety, and adherence of antihypertensive drugs in this study. It was also necessary to state the reason why the combination drug (a low-dose Triple Pill) was selected as the drug to be evaluated.<br><br>・The Methods section was well-described, but this reviewer thought they were too long. I thought some parts of the "Methods" section could be converted into supplementary material.<br><br>・In the Discussion section, the description of "healthy older adults" were inappropriate, because they had one or more cardiovascular risk factors.<br><br>・This reviewer felt that the title of this manuscript might not accurately represent the content of this manuscript.

VERSION 1 – AUTHOR RESPONSE

Reviewer: 1

1. Thank you for the opportunity to review this manuscript. This is a well-reported clinical trial and it is unfortunate that the study protocol was not feasible in this population, particularly because of the substantial evidence-practice gap in dementia prevention studies. The outcome of my review is detailed below.

Response: Thank you for your kind comments.

2. For the self-reported enrichment factors that indicated elevated risk of cognitive decline, how was the self-report information captured? Verbally? Or was written evidence required? E.g., verbal self-report of elevated LDL cholesterol seems unlikely.

Response: Cholesterol questions were captured during the 4-stage screening process. The first was self-reported as asked during stage 1 (online questionnaire) then as part of blood tests during stage 3. We have clarified this in the Methods section on page 8, paragraph 2:

"A 4-stage screening process was used (Figure 1) that consisted of: (i) an online questionnaire to assess initial eligibility *with questions on enrichment factors including elevated or high cholesterol*; (ii) telephone consultation for consent, and collection of demographic and clinical data, and a screening assessment using the Modified Telephone Interview for Cognitive Status (TICS-M),15,16 a 13-item telephone-based screening tool to detect pre-existing dementia or significant cognitive impairment; (iii) daily home BP monitoring using a certified OMRON device and the collection of fasting bloods for assessment of routine biochemistry (electrolytes and renal function), liver function, and *lipids*;"

3. Which of the recruitment techniques was the most effective? Some information here would be helpful for other researchers looking to recruit similar populations.

Response: We cannot reliably report that one approach was superior to another but a majority of participants were recruited through social media which was unlikely feasible as there were a large number of withdrawals and non contactable people. We have added a statement page 16, second paragraph:

“Our results do not clearly indicate a preferential approach to recruitment, they may also suggest that a broad range of recruitment techniques are required for such trials of prevention.”

4. For participants already receiving monotherapy treatment for hypertension, were there any medication changes in order to participate in the trial? Or did treatment as usual continue?

Response: Thank you for this important question. Patients were eligible in the study if they had moderately raised BP (SBP >120 and <160 mmHg or DBP > 80 and < 95 mmHg), whether or not they were on any antihypertensive treatment, or on treatment with a single agent at low to moderate dose. In order to participate in the trial, participants had to be taking a low or moderate dose to safely allow for the low dose triple study medication to be added to their treatment regime. Those who were taking an ACE-I, this had to be either stopped, or switched to an open label component of the triple pill such as telmisartan 20-40mg, indapamide 1.25mg, or amlodipine 2.5-5mg; or switched to a beta blocker. Information supplied in Supplementary Table 1.

5. RAVLT-D in the methods should be spelled out as RAVLT delayed recall.

Response: Thank you. This has now been corrected.

6. What safety criteria were used for AEs/SAEs and what framework was used for categorisation (severity, type)? There is detail in the supplementary materials, but a little more info needs to be included in the MS.

Response: An SAE form was available for completion in the event of SAE’s occurring in randomised participants. For this study, only two AESI’s were reported, one headache and one hyperkalaemia, there were no SAE’s reported. The SAE criteria were:

☐ Death

☐ Life-threatening

☐ Hospitalisation/prolongation of hospitalisation

☐ Significant disability/incapacity

☐ Congenital abnormality

☐ Other medically important event

The options for severity were mild, moderate and severe.

7. What did trial participants do at the end of the trial with any remaining medication? In the full protocol, it looks like it was returned to the pharmacy? Were participants compliant with this? Does this also mean that the medication was dispensed via participants’ local pharmacies? The success of these methods would help to inform future studies.

Response: Participants were sent medication from a central pharmacy associated with Syntro Health; participants were provided with stamped envelopes to send their medication bottles back to the central pharmacy. The majority of participants returned their study medication bottles and these were then destroyed by the pharmacy after accountability was undertaken.

8. For the linear mixed effects model, which factors were fixed and which were random?

Response: The model was specified with patient as the random effect, BP as the response, and fixed effects were mean BP, treatment, week(time) and an interaction between treatment and week(time). The methods section on page 13, paragraph 1 has been updated to:

“The overall BP difference between Triple-Pill and placebo was calculated using a linear mixed effects model, with post-randomisation BP as the dependent variable, fixed effects for baseline BP, treatment group, visit (week), and an interaction between the treatment variable and visit, and patient as a random effect.”

9. It would be worth noting the expected sample size in the main body of the manuscript and noting that the full justification is in the supplementary materials.

Response: Thank you for this comment, please see our response to the editor’s comment #3.

10. Trial registration # should be detailed in the MS. Was the protocol published?

Response: The trial registration was included in the abstract and now added under Methods, page 7, paragraph 1. The protocol has not been published but is included in the supplementary section of this manuscript.

11. How specific are the 49/125 screen failures associated with withdrawals or being non-contactable due to the recruitment methods used? The high level of screen failures at this point in the recruitment pipeline suggests that feasibility of the recruitment methods was low. Can the authors provide some additional discussion here please?

Response: Figure 2 outlines the screen failures of the recruitment steps. The majority of participants were screen failed between the online screening to stage 2 of the screening process. We agree that the recruitment methods may not have been ideal for this population. This has been discussed on page 16 as “Of 131 expressions of interest through social media, there were 125 screen failures which may reflect the non-targeted non-personal approach to participation (49 participants withdrew or did not answer when contacted) or use of an overly arduous, screening process.”

Further discussion has been added on page 16, paragraph 2:

“Recruitment of participants in the ATHENA study were through social media via TrialFacts and social media accounts on X (formerly Twitter) and Facebook. In addition, we performed a community campaign through a clinical trial registry and trial advertisement in primary care physician clinics. A majority of potential participants came through social media suggesting that while this type of recruitment campaign may initially capture the attention of the target population, it does not necessarily translate to commitment to participate in the trial as evidenced by 30 noncontactable and 8 withdrawal of potential participants after completing online screening (Figure 1).

12. In order to determine whether cognitive deficits interfere with ADLs, how were ADLs measured?

Response: ADLs were not measured; instead neuropsychological assessments for cognitive function were utilised as described in the Methods and protocol: Test of Premorbid Function, Preclinical Alzheimer’s cognitive composite and the Cogstate Brief Battery.

13. Given the lack of feasibility, the discussion could benefit a little more depth on what the researchers would do differently next time. Perhaps a ‘lessons learned’ or ‘recommendations’ section would be helpful to consolidate the key points (e.g., less screening? Different recruitment methods?).

Response: We have added recommendations on page 18, paragraph 3:

"We recommend a simplified one to two stage screening process to improve the recruitment of a trial using antihypertensive medications to attenuate cognitive decline in high-risk adults. Co-developing recruitment strategies with people with lived experience of cognitive decline and their caregivers likely will improve feasibility."

Minor:
14. Typos:
Page 8, Line 10 – 'lips' should this be lipids?
Page 8, Line 50 – Typo should be "complete the Patient…"
Page 9, Line 42 – Typo should be "began"
Supplementary materials, last line of exclusion criteria – Typo should be "psychiatric condition"

Response: Thank you for bringing these typos to our attention. They have been addressed.

15. Figure 2 has some slight formatting issues with arrows now being consistently aligned.

Response: This has been addressed.

Reviewer: 2

Summary

1. This study had so many variables to assess that it was difficult to understand the results. If possible, it may be possible to narrow down the evaluation variables in this paper and write other evaluation variables in separate papers.

Response: Thank you for this comment. We would prefer to present the full complement of results within a single paper so that the reader is aware of the full details of the recruitment together with summarising the vast amount of BP and cognition evaluation variables, and exit survey data, that were collected. However, we have removed 2 supplementary results BP change from baseline to randomised treatment (Supplementary table 4) and BP change from baseline trajectories over 4 week treatment period by randomised treatment (Supplementary figure 4).

2. How was the sample size calculated before the study started? The small sample size made interpretation of the results difficult.

Response: Thank you for this comment. Please see our explanation of the sample size of the study in relation to the Editors comments # 3.

3. In the introduction section, the authors should clearly state why there was necessary to evaluate the tolerability, safety, and adherence of antihypertensive drugs in this study. It was also necessary to state the reason why the combination drug (a low-dose Triple Pill) was selected as the drug to be evaluated.

Response: Thank you for this suggestion. We have added the following on page 6, second paragraph:

"A single, fixed low-dose, combination BP-lowering pill (a 'Triple Pill' containing telmisartan 20 mg, amlodipine 2.5 mg, and indapamide 1.25 mg) was selected as study medication as there is evidence that combination medications provide adequate BP control without adversely affecting the side effect profile. However, since the low-dose Triple pill has not been tested in older adults at increased risk of cognitive decline, we also aimed to determine the short-term tolerability, safety, and adherence to,

compared with matching placebo, in participants.”

4. The Methods section was well-described, but this reviewer thought they were too long. I thought some parts of the "Methods" section could be converted into supplementary material.

Response: Thank you for this comment. The Editor has suggested that we retain the full details in the Methods section.

5. In the Discussion section, the description of "healthy older adults" were inappropriate, because they had one or more cardiovascular risk factors.

Response: Thank you for your comment. We have now changed healthy older adults in the discussion to older adults with increased risk.

6. This reviewer felt that the title of this manuscript might not accurately represent the content of this manuscript.

Response: Thank you for your comment. We believe our title ‘A randomised controlled decentralised feasibility trial of a fixed low-dose combination antihypertensive drug strategy to attenuate cognitive decline in high-risk adults’ represent the trial’s key components (decentralised, feasibility trial, antihypertensive treatment to prevent cognitive decline).
